# A huge ovarian serous cystadenoma with situs inversus totalis: first case report

**DOI:** 10.1093/jscr/rjab065

**Published:** 2021-04-22

**Authors:** Erman Çetin, Eyüp Öner, Ulaş Aday, Mehmet Güzelgül, Ayça Orhan Gökçe

**Affiliations:** 1 Batman State Hospital, General Surgery Department, Batman, Turkey; 2 Department of Gastrointestinal Surgery, Dicle University School of Medicine, Diyarbakır, Turkey; 3 Maternity and Children Hospital, Batman, Turkey; 4 Batman State Hospital, Pathology Department, Batman, Turkey

**Keywords:** situs inversus totalis, ovarian serous cystadenoma, surgery

## Abstract

Coexistence of situs inversus totalis and ovarian serous cystadenoma in pubertal girls is extremely rare. It is important to preserve ovarian hormonal physiology and fertility if it is detected in the pubertal period.

A 16-year-old girl presented with abdominal distension and pain. Radiological evaluation revealed a huge abdominal cystic mass and situs inversus totalis. In laparotomy, unilateral salpingoophorectomy and total cystectomy were performed on the ovarian cystic mass. It was confirmed as serous cystadenoma in pathological evaluation.

This is the first reported case in the literature of situs inversus totalis with a huge ovarian serous cystadenoma.

## INTRODUCTION

Situs inversus totalis (SIT) is a very rare congenitalanomaly, which is characterized by the position of visceralorgans in the thoracic and abdominal cavity opposite to thatof normal human beings. It is a variation of anatomicalstructures of all internal organs due to visceral rotationdisorders during embryonic development [1]. Although serous cystadenoma and mucinous cyst adenoma are the most common benign epithelial ovarian tumors in the reproductive period, they are very rare in the pubertal period [2]. A case of huge mucinous cystadenoma and SIT has been presented in the literature, but there is no case of hugeovarian serous cystadenoma [1, 3].

We present a 16-year-old girl with SIT and a huge ovarian serous cystadenoma, which we think is the first in the literature.

## CASE REPORT

**Figure 1:**
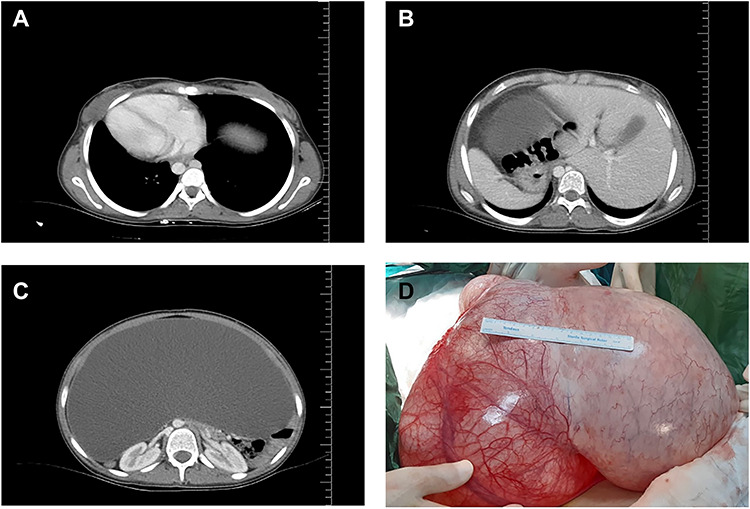
Organ locations (**A**, **B**) showing situs inversus totalis and axial (**C**) and coronal (**D**) views of the cystic mass on computed tomography.

**Figure 2:**
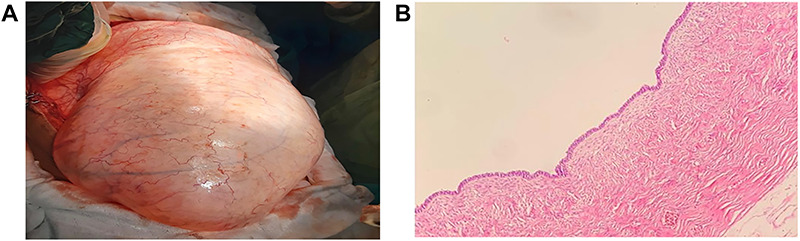
(**A**) Macroscopic view of the giant cystic mass taken out of the abdominal cavity. (**B**) The Cyst is lined by ciliated epithelium without significant nuclear atypia, H&E; x10.

A 16-year-old unmarried girl presented to our clinic with abdominal swelling and pain that had begun two years earlier and had been gradually worsening over the last one year. Increased abdominal distention was causing difficulty breathing. The patient had no known comorbidities, no prior surgeries, and no history of drug use. On physical examination, the abdomen was severely distended with a dull percussion in all quadrants. On cardiac auscultation, the heart beats were heard on the right side. Routine laboratory parameters, beta-human chorionic gonadotrophin (β-hCG),andtumor markers were normal. Abdominal ultrasonography (USG) showed diffuse free fluid in all quadrants. Contrast-enhanced thoracic and abdomino-pelviccomputed tomography (CT) visualized the heart on the right side and all other visceral organs on the opposite side and also revealed a 42x34 cm pure cystic giant mass extending from the pelvis to bilateral subdiaphragmatic spaces (Fig. 1). Based on these findings, the patient was diagnosed as having SIT. Following a multidisciplinary evaluation, a decision of laparotomy was made based on a prediagnosis of mesenteric, paraovarian, or ovarian cyst. Laparoscopy was not preferred due to the presence of dyspnea and the rupture risk of the cystic mass that was occupying the entire abdominal cavity. Surgical exploration during laparotomy indicated that the cyst originated from the right ovary, extending from the pelvis to the subhepatic region and neighbored by the anterior abdominal wall (Fig. 2). As the cyst originated from the right ovary, the left ovary was preserved and a right salpingo-oophorectomy was performed without any complications. The patient was discharged uneventfully on postoperative day 3. Pathological examination was reported as ovarian serous cystadenoma (Fig. 2). The patient has been followed up for six months and is still asymptomatic.

## DISCUSSION

Situs Inversus Totalis (SIT) is asymptomatic congenital anomaly with an unclear etiology. This anomaly occurs when embryonic midgut rotates 270° clockwise instead of rotating 270° counter-clockwise, leading to the placement of all the thoracic and abdominal visceral organs in symmetrical localization of the mid-line localization. In other words, SIT is the mirror image of the normal. Additionally, SIT has also been reported to be associated with a genetic defect occurring within the second week of embryonic life [4] and to be accompanied by bronchiectasia (Kartagener’s syndrome), polysplenia, genitourinary anomalies, andvarious organ cancers [5–8]. The incidence of tumors is higher in patients with visceral inversion. One possible hypothesis is that the expression of the tumor susceptibility gene has homology with abnormal chromosomes, though it has not been confirmed yet [1].

Ovarian/paraovarian cysts are rare cystic tumors in adolescents, commonly seen in the region among ovarian hilum, mesosalpinx, and ovarian fimbria within the broad ligament. These lesions are mostly benign, which account for 5–20% of all adnexal tumors and often originate from Mullerian (paramesonephric) or Wolffian (mesonephric) ductsorthe mesothelium. Giant ovarian cysts are often confused with mesenteric cysts, intraabdominal acid, peritoneal inclusion cysts, and lymphangiomas [9].

Patients with ovarian cysts often present with nonspecific symptoms such as abdominal pain, nausea, vomiting, and constipation. It is often difficult to distinguish these cysts, particularly giant cysts, from other abdominal pathologies and the diagnosis is usually made during laparotomy. USG is the method of choice in the diagnosis of ovarian cysts and also provides useful information in the differentiation of solid and cystic masses. Additionally, both CT and magnetic resonance imaging are highly useful in the differential diagnosis of intraabdominal masses as well as in the detection of additional pathologies and in surgical planning [1]. In the case presented, the origin of the cyst could not be elucidated by USG and CT and then the cyst was revealed to have an ovarian origin during laparotomy.

In general, the larger and more complex an ovarian lesion is, the more likely that it is a neoplastic process necessitating surgical removal. When a patient presents with an ovarian lesion measuring over 8 cm, the decision to proceed with surgical intervention is typically preferred over observation because larger lesions are less likely to resolve spontaneously, may be more prone to torsion, and are more likely to be malignant [10].

In conclusion, coexistence of SIT and giant ovarian serous cystadenoma is extremely rare and unusual. In such patients, a surgical technique that could eliminate the symptoms and the risk of malignancy after the identification of all anatomic variations associated with SIT would be the most ideal approach.

## CONFLICT OF INTEREST STATEMENT

The authors declare that they have no conflict of interest.

## GRANT SUPPORT & FINANCIAL DISCLOSURES

None.

## AUTHOR CONTRIBUTIONS

Conception and design of study: Çetin E, Aday U, Öner E.

Acquisition of data: Çetin E, Öner E, Güzelgül M, Gökçe AO.

Analysis and/or interpretation of data: Çetin E, Aday U, Gökçe AO.

Drafting the manuscript: Çetin, E, Öner E, Aday U, Gökçe AO.

Revising the manuscript critically for important intellectual content: Aday U, Çetin E, Güzelgül M, Gökçe AO.

Approval of the version of the manuscript to be published: Çetin E, Aday U, Öner E, Güzelgül M, Gökçe AO.
